# Factors influencing insulin prescribing practices among small animal specialists

**DOI:** 10.3389/fvets.2026.1792480

**Published:** 2026-05-21

**Authors:** Nicole E. DeRogatis, Katherine E. McCool, Cassie E. Donnell, Alex M. Lynch, Adam J. Birkenheuer

**Affiliations:** Department of Clinical Sciences, College of Veterinary Medicine, North Carolina State University, Raleigh, NC, United States

**Keywords:** clinical decision-making, diabetes mellitus, diabetic ketoacidosis, insulin, prescribing patterns

## Abstract

Initial management of diabetic ketoacidosis (DKA) often involves administration of regular insulin, and the transition from regular to intermediate or long-acting insulin can be challenging. While some guidelines exist for this, recent developments in the field have altered the diabetes treatment landscape. The objective of this study was to describe insulin prescribing practices of small animal specialists for patients with DKA and to explore the factors influencing the selection of insulin type and dose. An electronic survey involving two clinical case scenarios was distributed to diplomates of American College of Veterinary Internal Medicine (ACVIM) and American College of Veterinary Emergency and Critical Care (ACVECC) in Spring 2025. For the canine case, 76/162 participants (47%) selected insulin lente (Vetsulin^®^ or Caninsulin), with 74/162 (46%) selecting insulin lente administered twice daily (BID). The median dose for insulin lente BID was 0.43 U/kg/injection (range, 0.05–1.29 U/kg/injection). 62 out of 162 respondents (38%) selected insulin NPH (Humulin^®^-N, Novolin^®^-N) BID at a median dose of 0.43 U/kg/injection (range, 0.11–2.15 U/kg/injection). For the feline case, 120/151 participants (79%) selected insulin glargine 100 U/ml, with 118/151 (78%) selecting insulin glargine BID. The median dose for insulin glargine BID was 0.17 U/kg/injection (range, 0.04–1.87 U/kg/injection). The survey data describe current prescribing practices and clinical decision-making. As options for novel insulins and diabetic therapies expand, additional studies focused on outcomes related to clinical control, cost efficacy, and quality-of-life for pet and owner are needed.

## 1 Introduction

Diabetic ketoacidosis (DKA) is a common, life-threatening complication of diabetes mellitus (DM) in dogs and cats. Intravenous fluid therapy, correction of electrolyte abnormalities, and regular insulin are the mainstays of treatment for DKA patients (1). An important part of DKA and diabetic management involves the transition from a regular insulin protocol to an intermediate or long-acting insulin for outpatient management. This transition typically occurs when the patient is well-hydrated, tolerating oral feedings, has resolution of acid-base derangements, and has minimal to no ketones (1, 2). The transition from regular insulin to intermediate or long-acting insulin can be challenging and represents an important decision step in the management of this chronic disease.

The 2018 AAHA Guidelines for Diabetes Mellitus provide specific recommendations regarding insulin selection and dosing (3). However, since that time, recent developments in the field have altered the diabetes treatment landscape. Such changes include the discontinuation of insulin detemir (Levemir^®^), the decreased cost of the basal insulins degludec (Tresiba^®^) and glargine 300 U/ml (Toujeo^®^), new evidence supporting the utility of these insulins in dogs and cats (4–7), the increased popularity of flash glucose monitoring systems (FGMS) when managing dogs and cats with DM (8), and the generation of new guidelines surrounding diabetes management, including the iCat Care 2025 Consensus Guidelines (9). While the science of DM management is rapidly evolving, clinician preferences and practice styles regarding insulin therapies are unknown, highlighting the need to better understand clinicians' perspectives on insulin selection.

The objectives of this study were to describe insulin prescribing preferences of small animal specialists for patients with DKA and to explore the factors that influence selection of insulin type and dose.

## 2 Materials and methods

### 2.1 Survey design

The study was granted exemption by the NC State University Institutional Review Board (#27615). An electronic survey (Supplementary material) consisting of 10 multiple-choice, optional-response questions and 4 free-text, optional-response questions was developed to explore intermediate or long-acting insulin prescribing practices post-DKA management. The survey contained two cases, consisting of one dog and one cat with DKA. These two cases were based on real, newly diagnosed diabetic patients who were treated at NC State Veterinary Hospital for DKA and had complete medical record data available. Participants were provided with a summary of the case information, including signalment, presenting complaint, physical examination findings, laboratory data, serial blood glucose concentrations, and insulin constant rate infusion (CRI) data (Supplementary material). Based on this information, participants were asked to provide a specific insulin type, frequency, and dose (in units/injection) that they would recommend when transitioning the patient from a regular insulin CRI to an intermediate or long-acting insulin. Participants were also asked to rate the importance of 12 factors in selecting their initial dose of intermediate or long-acting insulin on a scale of 1 - “not important at all” to 5 - “very important.” For participants who selected “standard dose for initiation of insulin therapy” as an “important” or “very important” factor, follow-up questions were designed to determine when the standard was taught in their training and how consistently they apply this standard in practice. Prior to distribution, the electronic survey was evaluated by a subset of the authors, which included two board certified internists (KEM, AJB), one veterinary pharmacist (CD), and one small animal rotating intern (NED). The survey was also evaluated by three third-year internal medicine residents not involved in the survey design, who evaluated the survey for survey flow and user experience. Following minor edits, the survey was finalized.

While responses were anonymous, participants were asked to self-report demographic information, which included numbers of years in practice, type of veterinary practice, specialty type, and country of practice. To capture the potential presence of double boarded-clinicians within the survey pool, participants were provided with several choices of specialty from which to select [(ACVIM-SAIM, DACVECC, Other (*specify*)], and were instructed to select “all that apply”(Supplementary material).

### 2.2 Survey recruitment and distribution

Surveys were distributed using two distinct links to the following listservs 1) the American College of Veterinary Internal Medicine Small Animal Internal Medicine (ACVIM-SAIM) Diplomate listserv (*n* = 1,815) and 2) the American College of Veterinary Emergency and Critical Care (ACVECC) Small Animal Diplomate listserv (*n* = 1,017). The survey was available electronically during a four-week window from March 2025 to April 2025. Notification of survey availability consisted of one initial invitation and one reminder e-mail via each listserv.

### 2.3 Statistical analysis

Descriptive statistics (median, range) were performed on most survey measures. Because the answers were not “forced choice”, a different number of participants responded to each question, so when calculating response percentages, this was done on a per question basis. For the questions that asked for a dose of insulin in total units/injection for the canine and feline patients, the dose in “Units/kg/injection” was calculated by dividing the dose in units by the patient's body weight (kg). Responses were excluded if the participants did not list their dose in units or provided a dosing range instead of selecting a specific dose. Because specialty type was captured through both the survey link used and the self-reported demographic data, specialty classification was based on the survey link due to incomplete responses in the demographic section. Responses with discrepancies between these two sources were excluded from analysis of specialty type.

Comparative statistical analyses were performed using R (version 4.5.3; R Foundation for Statistical Computing, Vienna, Austria). A chi-square test of homogeneity was performed to evaluate the association between specialty type and insulin selection for each case scenario. For the canine case, because two insulin types (lente and NPH) were more frequently selected than others, all remaining insulin types (insulins degludec, glargine 300 U/ml, PZI, insulin glargine 100 U/ml, other) were grouped as “alternative insulin types”. For the feline case, because insulin glargine 100 U/ml was selected more frequently than other options, all remaining insulin types (insulin glargine 300 U/ml, PZI, lente, NPH) were grouped as “alternative insulin types”. Follow-up pairwise comparisons were performed using a Holm adjustment to account for multiple testing. A chi-square test of homogeneity was also performed to evaluate the association between practice type (academic vs. private for-profit practice) and insulin selection. The relationship between years in practice and selection of insulin was evaluated using two methods. Spearman's rank correlation coefficients were calculated to assess the association between years in practice and insulin selection for each case. Fisher's exact test was used to evaluate the association between years in practice group and insulin selection, without assuming an ordinal relationship between groups. Statistical significance was set at *P* ≤ 0.05.

### 2.4 Free-text response coding

Qualitative data analysis was performed by the corresponding author (KEM). These were extracted using inductive content analysis, a methodological approach for analyzing data in which themes are identified through the use of multiple rounds of systematic coding that allows overarching themes to emerge from the dataset without the use of predefined frameworks (10). Verification analysis was provided by the first author (NED) by reviewing the codes of the corresponding author and providing feedback and further refinement if needed. Once themes were established and confirmed by the first author (NED), frequency calculation was performed, which tallied the total number of times a code was used.

## 3 Results

### 3.1 Participants and demographics

A total of 162 individuals responded to at least one question on the survey; 79 (49%) responded using the ACVIM-SAIM link, and 83 (51%) responded using the ACVECC link. The response rates were 4% (ACVIM-SAIM) and 8% (ACVECC), respectively. No respondents reported dual board certification. One respondent accessed the survey via the ACVIM-SAIM listserv but self-identified as a DACVECC in the demographic section; this response was excluded from analyses evaluating the association between specialty type and insulin selection. Not every individual responded to every question on the survey, so the total number of responses varied between questions. Complete demographic data are presented in Table 1. At the time of the survey, 79% of participants (*n* = 103/131) were working in for-profit private practice, 12% (*n* = 16/131) were working in academia, 5% (*n* = 6/131) were working in non-profit private practice, and 5% (*n* = 6/131) were working in a setting characterized as “other.” 60 percent of participants (*n* = 78/131) had been practicing veterinary medicine for 15 years or fewer. 90 percent of participants were practicing in the United States (*n* = 118/131), 6% (*n* = 8/131) were practicing in Canada, and one participant was practicing in each of the following locations: France, the United Kingdom, the United States/West Indies, Australia, and an unspecified country at the time of the survey.

**Table 1:** Demographic data of participants.

Category		*n*	% of participants
Diplomate status	DACVIM	79	49
	DACVECC	83	51
	Total	162	–
Years in practice	1–5	3	2
	6–10	36	28
	11–15	39	30
	16–20	19	15
	21–25	19	15
	26–30	9	7
	31–35	3	2
	36–40	1	< 1
	41–45	1	< 1
	>46	1	< 1
Country	United States	118	90
	Canada	8	6
	France	1	< 1
	United Kingdom	1	< 1
	United States and West Indies	1	< 1
	Australia	1	< 1
	Other	1	< 1
Practice type	For-profit private practice	103	79
	Academic institution	16	12
	Non-profit private practice	6	5
	Other	6	5
	Total	131	–

Diplomate status (n = 162) was based on the survey link due to incomplete responses to questions in the demographic section of the survey.

Demographic information regarding years in practice, country, and practice type was based on responses to the questions in the demographic section of the survey (n = 131).

### 3.2 Participant responses: insulin type and dose

All 162 participants responded to the insulin-selection question for the canine case scenario, and (*n* = 151/162; 93%) responded to the insulin selection question for the feline case scenario. For the two respondents who indicated that they would select “Caninsulin^®^” for either case, the responses were included under the insulin lente (Vetsulin^®^) responses. Responses from 16 participants (*n* = 16/162; 10%) for the canine case and six participants (*n* = 6/151; 4%) for the feline case were excluded from dosing analyses because doses were reported in units/kg or as ranges rather than as a single value.

### 3.3 Selection of insulin type, dose, frequency: canine case

Seventy-six of 162 participants (76/162; 47%) selected insulin lente (Vetsulin^®^ or Caninsulin), with 74 of 162 (*n* = 74/162; 46%) selecting insulin lente administered twice daily (BID). The median dose for insulin lente BID was 0.43 U/kg/injection (range, 0.05–1.29 U/kg/injection). 62 of 162 participants (*n* = 62/162; 38%) selected insulin NPH (Humulin^®^-N, Novolin^®^-N) BID at a median dose of 0.43 U/kg/injection (range, 0.11–2.15 U/kg/injection). 11 participants (*n* = 11/162; 7%) selected insulin degludec, five (*n* = 5/162; 3%) selected insulin glargine 300 U/ml (Toujeo^®^), four (*n* = 4/162; 2%) selected protamine zinc insulin (PZI) (ProZinc), two (*n* = 2/162; 1%) selected insulin glargine 100 U/ml, and two (*n* = 2/162; 1%) selected “other”. The two participants that selected “other” did not select a specific insulin type and clarified their choice of insulin selection in the free-text box. One described the use of Vetsulin^®^ pending the preferences of the primary care veterinarian while another chose a combination of insulins.

22 participants out of 162 (*n* = 22/162; 14%) selected a dose that was in-between full-unit increments. Among these, 17 (*n* = 17/162; 10%) chose a dose in half-unit increments, and five (*n* = 5/162; 3%) chose a dose smaller than a half-unit (for example, 1.25 or 1.2 units/injection). A summarized list of participant responses, including insulin types, frequency of administration, and descriptive statistics regarding the dose per injection (U/kg/injection), are presented in Table 2.

**Table 2:** Selection of insulin type, dose, frequency: canine case.

Type of insulin	*n*	(% of participants)	Frequency of administration	Total	(% of participants)	Median dose (U/kg/injection)	Min (U/kg/injection)	Max (U/kg/injection)
Lente (Vetsulin^®^)	76	47	SID	2	1	0.16	0.11	0.22
			BID	74	46	0.43	0.05	1.29
NPH (Humulin^®^ N, Novolin^®^ N)	62	38	SID	0	–	–	–	–
			BID	62	38	0.43	0.11	2.15
Degludec (Tresiba^®^)	11	7	SID	7	4	0.43	0.22	0.65
			BID	4	2	0.43	0.22	0.65
Insulin glargine 300 U/ml (Toujeo^®^)	5	3	SID	4	2	0.43	0.43	0.65
			BID	1	1	0.22	0.22	0.22
PZI (ProZinc^®^)	4	2	SID	0	–	–	–	–
			BID	4	2	0.54	0.43	0.86
Insulin glargine 100 U/ml	2	1	SID	0	–	–	–	–
			BID	2	1	0.32	0.22	0.43
Other	2	1	–	–	–	–	–	–
Total	162	–	–	–	–	–	–	–

SID, once daily administration; BID, twice daily administration.

Participants that selected “other” did not select a specific insulin type and clarified their choice of insulin selection in the free-text box.

There was an association between specialty type and insulin selection (χ^2^ (2) = 21.00, *P* < 0.001). ACVECC diplomates were more likely than ACVIM-SAIM diplomates to select NPH (χ^2^(1) = 12.91, *P* < 0.001) and were less likely to select one of the alternative insulin types (χ^2^(1) = 12.15, *P* < 0.001). There was no association between specialty type and the selection of insulin lente; χ^2^(1) = 0.72, *P* = 0.397. There was no association between insulin selection and practice type (χ^2^(2) = 2.71, *P* = 0.258). Spearman's correlation showed no correlation between years in practice and selection of insulin lente (ρ = 0.073, *P* = 0.40) or NPH (ρ = −0.017, *P* = 0.85). Fisher's exact test revealed no significant association between years in practice and insulin selection, regardless of the ordering of the groups (*P* = 0.82).

### 3.4 Selection of insulin type, dose, frequency: feline case

120 of 151 participants (*n* = 120/151; 79%) selected insulin glargine 100 U/ml, and 118/151 (78%) selected insulin glargine 100 U/ml BID. The median dose for BID administration was 0.17 U/kg/injection (range, 0.04–1.87 U/kg/injection). 13 of 151 participants (*n* = 13/151; 9%) selected insulin glargine 300 U/ml (Toujeo^®^), 10 (*n* = 10/151; 7%) selected PZI, five (*n* = 5/151;3%) selected insulin lente, and three (*n* = 3/151; 2%) selected NPH. 12 of 151 participants (*n* = 12/151; 8%) selected a dose of insulin in-between full-unit increments; nine (*n* = 9/151; 6%) chose a dose in half-unit increments, and three (n = 3/151; 2%) chose a dose smaller than a half-unit. A summarized list of participant responses, including insulin types, frequency of administration, and descriptive statistics regarding the dose per injection, are presented in Table 3.

**Table 3:** Selection of insulin type, dose, frequency: feline case.

Type of insulin	*n*	(% of participants)	Frequency of administration	Total	(% of participants)	Median dose (U/kg/injection)	Min (U/kg/injection)	Max (U/kg/injection)
Insulin glargine 100 U/ml	120	79	SID	2	1	0.34	0.34	0.34
			BID	118	78	0.17	0.04	1.87
Insulin glargine 300 U/ml (Toujeo^®^)	13	9	SID	4	3	0.51	0.51	0.51
			BID	9	6	0.43	0.17	0.51
PZI (ProZinc^®^)	10	7	SID	0	–	–	–	–
			BID	10	7	0.34	0.04	1.02
Lente (Vetsulin^®^)	5	3	SID	0	–	–	–	–
			BID	5	3	0.38	0.17	0.51
NPH (Humulin^®^ N, Novolin^®^ N)	3	2	SID	0	–	–	–	–
			BID	3	2	0.60	0.34	0.85
Total	151	–	–	–	–	–	–	–

SID, Once daily administration; BID, twice daily administration.

There was no association between specialty type and insulin selection (χ^2^(1) = 0.24, *P* = 0.627) or between practice type and insulin selection (χ^2^(1) = 0.01, *P* = 0.903). Spearman's correlation showed no correlation between years in practice and selection of insulin glargine 100 U/ml (ρ = −0.05, *P* = 0.61,). Fisher's exact test revealed no significant association between years in practice group and selection of insulin glargine 100 U/ml, regardless of the ordering of the groups (*P* = 0.302).

### 3.5 Self-reported factors influencing selection of insulin type, dose, and frequency

**Figure 1:**
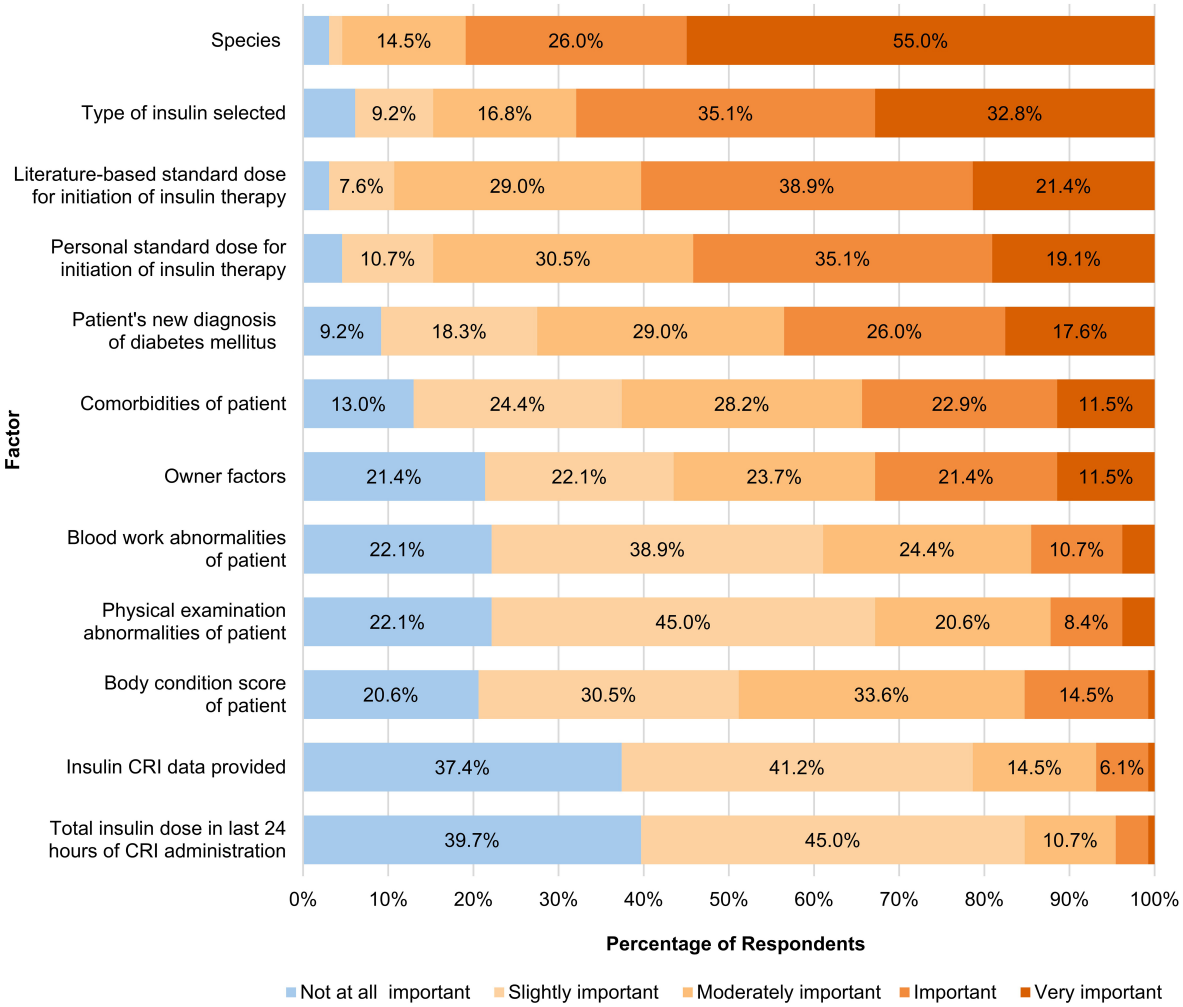
Factors influencing selection of insulin type, dose and frequency (*n* = 131). Categories with values less than 5% are not labeled.

Factors influencing selection of insulin type and dose are presented in Figure 1. The factors most frequently considered either “important” or “very important” were species (81%), type of insulin selected (68%), and literature-based standard dose (60%) for initiation of insulin therapy.

In addition to selecting from a pre-populated list of factors, participants were also asked to provide free text comments related to other factors influencing selection of insulin. A total of 42 free text comments were noted. Thematic analysis of free-text comments identified themes related to cost and owner finances (*n* = 9), in-hospital patient biochemical trends (*n* = 7), patient appetite (*n* = 6), patient factors (*n* = 5), insulin characteristics (*n* = 4), clinician preference (*n* = 3), primary care veterinarian preferences (*n* = 3), risk of hypoglycemia (*n* = 2), diabetic monitoring strategy (*n* = 2), and miscellaneous (*n* = 1). A copy of all free-text responses on this question and their corresponding themes is included (Supplementary material).

### 3.6 Factors impacting personal standard for initiation of insulin therapy

71 of 131 participants (*n* = 71/131; 54%) rated the use of a “personal standard starting dose” for initiating insulin therapy as “important” or “very important.” Among this subset (*n* = 71), participants reported that they applied this standard dose in practice 31%−100% of the time (median 81%). Participants were also asked to identify at which point(s) in their training this standard was developed. 37 of 71 participants (*n* = 37/71; 52%) selected three or more time points in their training during which this standard was developed. The two most common timepoints identified were during residency from advice assimilated from multiple mentors (*n* = 49/71; 69%) and post-residency from personal experience (*n* = 34/71; 48%). A summarized list of participant responses is presented in Table 4.

**Table 4:** If you rated “personal standard dose for initiation of therapy” as “important” or “very important”, at what point in your training was this standard taught? (Select all that apply).

At what point in your training was this standard taught?	Number of participants	Percentage (%)
During residency from advice assimilated from multiple mentors	49	69
Post-residency from personal experience	34	48
During internship from advice assimilated from multiple mentors	24	34
Learned from literature	23	32
During residency from personal experience	22	31
Post-residency from advice assimilated from multiple mentors	22	31
During veterinary school from personal experience in clinical rotations	10	14
During internship from personal experience	10	14
During veterinary school as part of curriculum	6	8
During residency from a single mentor	4	6
Other (specify)	2	3
Post-residency from a single mentor	2	3
During internship from a single mentor	1	1

Participants (*n* = 71) were permitted to select multiple responses. Values are reported as number (n) and percentage (%) of participants endorsing each statement.

### 3.7 Additional comments

A final survey question asked participants to share any additional comments, and 10 participants (*n* = 10/162; 6%) responded to this question. Thematic analysis of the free-text comments revealed themes related to a transition to an intermediate or long-acting insulin (*n* = 4/10), a desire for more clear, evidence-based guidelines regarding insulin dosing (*n* = 2/10), in-hospital management of DKA (*n* = 2/10), and feedback on the survey itself (*n* = 2/10). A copy of all free-text responses on this question and their corresponding themes is included (Supplementary material).

## 4 Discussion

The objective of this study was to describe insulin prescribing preferences of small animal specialists for patients with DKA and explore the factors influencing these choices. We found that insulin lente BID and insulin NPH BID were the top two choices for the canine case, and insulin glargine 100 U/ml BID was the top choice for the feline case. ACVECC diplomates were more likely to select NPH for the canine case and were less likely to choose one of the alternative insulins (an insulin other than NPH or lente). Self-reported factors influencing insulin selection included: species, type of insulin selected, and literature-based dose for initiation of insulin therapy.

For the canine case, the median starting dose of insulin lente BID was 0.43 U/kg/injection (range, 0.05–1.29 U/kg/injection). While the first choice of insulin lente is consistent with the recommendations of the 2018 AAHA Diabetes Management Guidelines for Dogs and Cats, the median dose selected was slightly higher than the 0.25 U/kg BID recommended by these guidelines (3). Insulin NPH BID was also a popular choice, selected by 38% of participants, with a median dose per injection of 0.43 U/kg/injection (range, 0.11–2.15 U/kg/injection), which is within the range provided by the AAHA DM Guidelines for NPH in dogs (0.25–0.5 U/kg/injection BID) (3). Insulin lente and insulin NPH are both intermediate-acting insulin options with decades of clinical use and multiple studies demonstrating their efficacy (4, 11). Other options, such as insulin degludec and insulin glargine 300 U/ml, were less frequently chosen by participants. These insulins, which have recently become economically feasible for use in veterinary practice, have less day-to-day variability compared with other insulin types (4–6). This reduced variability with basal insulins makes dosing and diabetic regulation easier as rapid insulin dose titration with FGMS can be performed (5, 6). In addition, these insulins are frequently administered once daily instead of twice daily (5, 6), which contrasts with traditional insulin dosing in dogs with insulin lente and insulin NPH, and may allow for greater owner flexibility. The relatively low numbers of participants selecting insulin degludec or insulin glargine 300 U/ml may be explained by the timing of our survey distribution. Our survey was distributed to the ACVIM-SAIM and ACVECC-SA listservs in Spring 2025, only several months following the publication of the first reports of the use of insulin glargine 300 U/ml (5) and insulin degludec (6) in client-owned diabetic dogs. As the veterinary community gains increasing experience with these insulins and additional evidence is generated, these insulins may become more popular, and guidelines from veterinary organizations such as AAHA should be updated to provide guidelines around selection of these long-acting basal insulin formulations. For the feline case scenario, insulin glargine 100 U/ml administered twice daily was the most frequently-selected insulin regimen, at a median dose of 0.17 U/kg/injection, or approximately 1 unit/cat. For cats with diabetes, the AAHA guidelines recommend initiating insulin therapy with glargine 100 U/ml or PZI at a starting dose of 1–2 units per cat q12 h (3). Although the AAHA guidelines recommend PZI as a first-line option for cats with diabetes, this choice was only selected by 7% of participants. Potential reasons for this include personal preference, cost, or prior evidence demonstrating that newly diagnosed diabetic cats treated with glargine 100 U/ml had better glycemic control and a higher probability of remission compared to cats treated with PZI or lente insulins (12). Other more recent guidelines, the iCat Care 2025 Guidelines on the Diagnosis and Management of Diabetes Mellitus in Cats, published after our survey was performed, do not recommend a specific insulin type for cats, instead emphasizing that the choice of insulin for an individual cat depends on multiple factors, including diet, pet owner goals, compliance, monitoring strategies, and caregiver lifestyle/concerns (9).

Interestingly, clinician selections of dosing and insulin type did not always align with official label recommendations. This observation may be related to discrepancy between the labeled dose of some drugs and the current expert recommendations regarding use. For example, while the AAHA Guidelines recommend a starting dose for insulin lente of 0.25 U/kg subcutaneously twice daily in dogs (3), Vetsulin^®^'s official label recommends an initial once-daily dose of “0.5 units insulin/kg” and that “twice daily therapy should be initiated if the duration of insulin action is determined to be inadequate” (13). In the canine scenario, most participants selected twice daily dosing, with only two selecting once daily dosing. Additionally, while PZI is labeled for use in dogs, it was selected by a minority of participants. PZI has been noted to have significant variability in its time course of insulin action (14), which can make for a less predictable glycemic response (15, 16).

Approximately 14% of participants for the canine case and 8% of participants for the feline case selected an insulin dose rounded to an increment less than 1 unit. This is important because research has shown that use of a syringe to draw up very small volumes of insulin at one unit or less may result in imprecise dosing (17) and the current AAHA guidelines recommend rounding to the nearest unit when calculating doses (3). While several of the insulins in our study (degludec, glargine 100 U/ml, glargine 300 U/ml) come in pen form, we did not specifically ask about method of insulin administration (administration from pen directly vs. drawing insulin out of pen device and administering with a syringe), and this represents a study limitation. Studies from human patients have shown that insulin pens offer multiple advantages over the traditional syringe administration method in terms of accuracy (18, 19), adherence (18, 20, 21), and patient satisfaction (18).

A second objective of our study was to explore the factors influencing the selection of insulin dosage and type. Top factors influencing the selection of insulin therapy included: species, type of insulin, and the literature-based standard. Approximately 60% of clinicians indicated that the literature-based standard was an “important” or “very important” factor in their insulin decision-making. While various resources exist to guide clinicians in insulin dosing and selection (3, 13) and in describing initial treatment and monitoring protocols for outpatient insulin therapy (5, 6), there are no specific resources to our knowledge that outline the recommendations to transition from a regular insulin CRI to subcutaneous insulin choices in DKA patients. Anecdotally, some clinicians will advocate for the use of the in-hospital dose to estimate the at-home dose, but no peer reviewed veterinary studies have explored this method. Studies in human DKA patients have shown that 60%−70% of the 24-h insulin infusion requirement as the subcutaneous dose of insulin resulted in glycemic control for 70% of patients (22). Similarly, a dose equal to 80% of the total daily insulin requirement, calculated from the average rate in the last 6 h of the infusion, was found to provide the highest percentage of patients with glycemic control within the first 24 h of the transition, compared to groups receiving 40 and 60% (23). Another protocol used a method of calculating the total daily dose of insulin required by averaging the infusion rate of the previous 6 h and multiplying that by a coefficient related to whether the patient was taking in full or minimal nutrition (24). In this study, known diabetics following the protocol obtained better glycemic control than those without a specified protocol (24). It is interesting to note that two of the participants in the free-text comments specifically identified the desire for clearer, evidence-based guidelines regarding dosing in switching from a DKA protocol to intermediate or long-acting insulin. This highlights a knowledge gap that warrants further investigation.

For the canine case, we found that ACVECC diplomates were more likely to choose NPH, and less likely to choose “alternative insulin” including degludec and glargine 300 U/ml. There may be several different reasons for this. Initial monitoring for insulin degludec and insulin glargine 300 U/ml typically includes monitoring with a FGMS to allow for rapid up-titrations of doses (5, 6). It is not surprising that critical care specialists, whose focus is on immediate patient stabilization and acute care, may opt to start a patient on an intermediate insulin like NPH and avoid insulins which require more intensive initial follow-up monitoring and owner communication.

In our survey, only 33% of participants found owner factors to be “important” or “very important” for decision-making related to insulin selection. One reason for this finding may be due to the nature of a survey study. While these cases were taken directly from the medical record from real cases at our institution, many clinical decisions are dynamic in nature and are a collaboration between the owner, specialist, and primary care veterinarian. Owner considerations are a critical part of DM management (9, 25) and include factors such as the capacity to give insulin once or twice daily, ability to pair insulin injection with meals, preferred diet types, overall goals of care, and monitoring preferences. Monitoring preferences may include low-intensity monitoring (such as use of clinical signs alone) to more intensive monitoring (use of FGMS in conjunction with clinical signs). A survey-based study may not have provided sufficient context to mirror real-life clinical decision-making. Additional future studies could incorporate more dynamic ways to assess insulin prescribing practices and decision making (such as focus groups) or could directly study actual prescribing patterns of large groups of specialists and primary care veterinarians. There is currently little published evidence that one insulin formulation is superior to another for diabetic treatment, so an emphasis on affordability, predictability, and client, patient, and veterinarian comfort is of utmost importance (9, 25). It is interesting to note that many of our free-text comments reflected this, with many comments on decision making related to cost and owner finances, patient factors (including appetite, concurrent medications, and diabetic monitoring strategy), and primary care veterinarian factors. As the science of DM management advances and a more diverse array of treatments become available, the field is shifting away from the idea of a “best insulin” toward a focus on therapy tailored to the specific patient and owner needs (9, 25).

In addition to these limitations, our survey was limited to two case scenarios, and we did not ask about wider prescribing patterns. This limitation could have prohibited us from seeing the full spectrum of responses that may have been captured in a survey with more clinical case scenarios or a survey focused on general prescribing patterns. Other factors that may limit the generalizability relate to the population of participants that responded to our survey. Response rates from each listserv were relatively low (4 and 8%), and this small group may not be representative of the overall population of internists and criticalists in practice who treat patients with DKA. There may have been inherent nonrespondent bias in this study, which may have skewed the results to represent specialists that are more interested in treating diabetes mellitus rather than the general population of specialists. Though respondents had the opportunity to select multiple specialties in the demographic section, there were no measures in place to ensure a diplomate on both listservs did not take the survey twice. The majority of our study participants (79%) worked in for-profit private practice, which closely mirrors recent survey data from the ACVIM and ACVECC that showed that 67% of ACVIM-SA diplomates and 72% of ACVECC diplomates work in private practice (26, 27). While 96% of participants practiced in the United States and Canada, the remainder practiced outside of the United States. This is important because the cost and availability of certain insulins may vary widely across the globe, and some countries, such as the United Kingdom, require the use of a licensed veterinary product if one exists, which would require prescribers to use the veterinary insulins (Vetsulin^®^ or Caninsulin^®^) or PZI^®^. Other limitations of this study relate to nuances of the survey design, particularly question wording. Specifically, two questions asked participants to report an insulin dose in “units per injection,” rather than “units/kg,” to better reflect real-world decision making in which clinicians must commit to a specific starting dose. A small subset of participants (9% for the canine case and 4% for the feline case) reported doses in units/kg or provided a dosing range, leading to exclusion of these responses from the dosing analysis. This pattern suggests that the dosing question may have been unclear to some participants, which could have influenced their responses. Future studies should ensure clearer wording of dose-related questions. A final limitation of this study relates to the fact that we focused solely on the role of insulin therapy, which constitutes one of many aspects of global diabetic management. Other important factors in the treatment of DM include diet (3), monitoring strategies, and other additional therapies such as SGLT-2 inhibitors (28, 29).

Our study focused on insulin prescribing practices and clinical decision-making. However, future outcomes-based research is needed. While some recent studies have evaluated the pharmacodynamics and pharmacokinetics of different insulin formulations in healthy dogs (30) and the day-to-day variability of various insulins in research dogs with DM (4), there have been few studies directly comparing clinical outcomes using different types of insulins in patients with DM (12, 31). As our options for insulins expand, additional prospective studies evaluating insulins with respect to glycemic control and quality-of-life for pet and owner are needed. These studies could include metrics such as time to clinical control using FGMS data, Diabetes Alive Clinical Scores (32), and validated owner questionnaires (33).

The results of this survey using two clinical case scenarios showed that insulin lente and insulin NPH administered twice daily for dogs and insulin glargine 100 U/ml administered twice daily for cats were the most popular insulin therapies. These selections generally align with current treatment recommendations (3). While less common, participants also selected insulin degludec and insulin glargine 300 U/ml, two insulins with recent data on use in client-owned patients (5–7). As the range of available insulin options continues to expand, prescribing practices may evolve as additional outcome data emerges.

## Data Availability

The original contributions presented in the study are included in the article/Supplementary material. Further inquiries can be directed to the corresponding author.
