# Supplementary material for: Factors influencing insulin prescribing practices among small animal specialists
Source: Front Vet Sci. 2026 May 21;13:1792480. doi: 10.3389/fvets.2026.1792480 (PMC13233279; doi:10.3389/fvets.2026.1792480)
Supplement: Supplementary file 1 [file Table_1.docx]

**Case 2:**Bart

Bart, a 4-year-old male neutered Domestic Shorthair, was presented to a tertiary referral hospital for newly diagnosed diabetes mellitus, DKA and hyporexia.

**Weight:**5.88 kg
**Vitals on presentation:** T 101.0F, P 190, R 30
**Physical examination abnormalities:** quiet to dull mentation, ~5% dehydrated

Initial blood work revealed a blood glucose level of 324 mg/dL (normal = 69-149 mg/dl), blood ketone levels of 4.4 mmol/L (normal = <2.55 mmol/L), blood pH of 7.09 (normal = 7.24-7.40), and blood bicarbonate levels of 11.2 mmol/L (normal = 15.6-26.5 mmol/L), confirming that Bart was in DKA. A regular insulin CRI was initiated at 0.05 U/kg/hr.

Additional information regarding Bart's case can be found at the following link: [Bart](https://ncsu.qualtrics.com/CP/File.php?F=F_3BEx94tCoK2i7zg)

Bart was hospitalized for ongoing management of DKA. His regular insulin CRI was discontinued with an average blood glucose level of 280 mg/dL in the last 6 hours of administration.

In the last 24 hours of regular insulin administration prior to discontinuation, his average insulin CRI rate was 0.2 U/kg/hr. His total daily insulin dose in the last 24 hours of administration was 5 U/kg, which equates to approximately 29 U total.

Bart – Additional Information

History:

Bart, a 4-year-old male neutered Domestic Shorthair, was presented to a tertiary referral hospital for evaluation of a 1-month history of progressive hyporexia to anorexia starting 6 days prior to presentation. His past medical history includes intermittent upper respiratory infection signs, suspected to be secondary to feline herpesvirus.

Current medications: None

Diet: Fancy Feast wet and dry (unknown daily amount) Lifestyle: Indoor only

Full physical examination on presentation:

**Weight:** 5.88 kg

**Vitals:** T 101.0F, P 190 bpm, R 30 bpm

**General appearance:** QAR to dull. Estimated 5% dehydration.

**Ophthalmic:** Eyes mildly sunken OU. Mydriatic but responsive pupils OU. Cornea/anterior chamber clean/clear OU. Mildly icteric sclera OU. No ocular discharge.

**Otic:** Vertical canal contains scant brown waxy debris. No obvious erythema or pain on palpation of external canal.

**Nasal:** Normal nasal architecture with no nasal discharge.

**Oral:** Mild to moderate generalized periodontal disease. Moderate gingivitis most prominent caudal molars. No oral masses or ulcerative lesions.

**Cardiovascular:** No murmur or arrhythmia. Fair, synchronous femoral pulses. Pale pink mucous membranes. CRT 1-2 seconds.

**Respiratory:** Normal bronchovesicular sounds in all lung fields. No crackles or wheezes. Eupneic.

**Abdomen/gastrointestinal:** Soft, non-painful on abdominal palpation. Slightly doughy small intestines diffusely. No palpable mass effect, fluids wave or organomegaly.

**Urogenital:** Normal external anatomy. Neutered male.

**Musculoskeletal:** Ambulatory with no obvious lameness x4. Mild generalized muscle wasting (MMI 2.5/3). Overconditioned (BCS 7/9).

**Integumentary:** Dry fur coat with scaling along dorsum. Left cephalic catheter in place secured with vet wrap. Right forelimb shaved.

**Lymphatic:** Superficial cervical, submandibular and popliteal lymph nodes are small, soft and symmetrical. Axillary and inguinal lymph nodes not palpable.

**Neurologic:** QAR but otherwise appropriate mentation. Cranial nerves normal (PLR, menace, palpebral, facial sensation, VOR, no positional strabismus or nystagmus). Full neurologic assessment not performed.

**Rectal:** Not evaluated.

Initial diagnostics on presentation:

1. Point-of-care blood work: BG 324, PCV 32%, TS: 9.0

1. Blood ketones: 4.4 mmol/L
2. ECG: HR 166, normal sinus rhythm
3. Venous blood gas: pH 7.09, pCO2 33, pO2 49, Na 129, K 3.2, Cl 96, iCa 1.13, Glu

246, Lac 2.5, TCO2 12.2, BE -16.6, Bicarb 11.2, Hct 27

1. Complete blood count: WBC 21.75 (H), HCT 24.7% (L), MCV 43.9, MCHC 41.2 (H),

Retic abs 37,000, Segs 19.358 (H), Lymph 0.218 (L), Mono 2.175 (H), Lymph 0.218 (L), PLT 488K (H), PP 7.5

1. Chemistry panel: Glu 188 (H), BUN 10 (L), Creat 0.5 (L), Phos 1.8 (L), Ca 7.6 (L), Mg

1.2 (L), TP 5.7 (L), Alb 3.2, Glob 2.5 (L), Chol 300 (H), Tbili 6.5 (H), ALP 40, ALT 204

(H), AST 172 (H), GGT <3, CK 1036 (H), Na 134 (L), K 4.2, Cl 95 (L), Bicarb 8 (L), AG

35.2 (H), Amy 421 (L), Lip 19

1. Abdominal ultrasound conclusions:
   1. Hyperechoic hepatomegaly-most compatible with hepatic lipidosis given the clinical history; concurrent underlying vascular hepatopathy associated with known endocrinopathy is also potentially contributing to this appearance.
   2. Bilaterally large kidneys with otherwise normal renal parenchyma. This can occur in male cates secondary to fat deposition and may be nonpathological; however concurrent round cell neoplasia such as lymphoma may also be possible
   3. Diffuse small intestinal muscularis thickening-consider inflammatory bowel disease or enteritis as well as infiltrative round cell neoplasia such as lymphoma.

4. Echogenic cholecystic debris.

Initial therapeutics given in hospital:

1. IV fluids: 0.45% NaCl 40 mL/kg/day, LRS to correct for 5% deficit over 24 hours

1. IV fluid additives: KCl 0.05 mEq/kg/hr, KPhos 0.1 mEq/kg/hr
2. Regular insulin CRI 0.05 U/kg/hr
3. Gabapentin 10 mg/kg PO q8h
4. Pradofloxacin 7 mg/kg PO q24h
5. Maropitant 1 mg/kg IV q24h
6. Ondansetron 0.5 mg/kg IV q8h

8. Nasogastric tube placement and subsequent feedings

*Graph 1: Patient’s regular insulin CRI rates and respective blood glucose and blood ketone levels in the final 24 hours of regular insulin CRI administration. Patient was initially started on a regular insulin CRI of 0.05 U/kg/hr at the start of hospitalization (not included on this graph).*
