# Supplementary material for: Factors influencing insulin prescribing practices among small animal specialists
Source: Front Vet Sci. 2026 May 21;13:1792480. doi: 10.3389/fvets.2026.1792480 (PMC13233279; doi:10.3389/fvets.2026.1792480)
Supplement: Supplementary file 2 [file Table_2.docx]

**Supplementary material.** Free-text comments in response to the question, “Please rate the importance of any additional factors (not listed above), on a scale from “1-not at all important-1” to “very-important-5”; (n=42).

| **Please rate the importance of the following factors on a scale from "not at all important-1"  to "very important-5".** | **Comment** | **Code or Theme** |
| --- | --- | --- |
| 4 | Owner budget | Cost and owner finances |
| 5 | Basal insulin | Insulin characteristics |
| 5 | Cost of the insulin | Cost and owner finances |
| 4 | Cost of insulin if owner expressed financial hardship | Cost and owner finances |
| 4 | BG at presentation | In-hospital patient biochemical trends |
| 4 | Clinical experience with said insulin | Clinician preference |
| 5 | Cost | Cost and owner finances |
| 4 | relative risk (rather under-dose than over-dose) | Risk of hypoglycemia |
| 5 | Cost (of insulin) | Cost and owner finances |
| 3 | Large vs small breed dog | Patient factors |
| 4 | Pen form available | Insulin characteristics |
| 5 | Eating habits of pet (grazing, meal feeding) | Patient appetite |
| 5 | whether or not the patient is going home with an interstitial glucose sensor on at discharge | Diabetic monitoring strategy |
| 5 | how well eating | Patient appetite |
| 4 | whether patient is receiving medication that could cause insulinoresistance ie. steroids | Patient factors |
| 3 | Steroid administration increasing initial insulin needs of the dog | Patient factors |
| 5 | Estimated Cost per pet per month | Cost and owner finances |
| 4 | Resolution of ketones prior to starting long/intermediate acting insulin | In-hospital patient biochemical trends |
| 3 | Rdvm in area tend to have strong preferences for insulin type in their patients | Primary care veterinarian preferences |
| 5 | experience | Clinician preference |
| 5 | Response in hospital for next 12-24 hours of initial dose of insulin chosen | In-hospital patient biochemical trends |
| 3 | blood ketone levels | In-hospital patient biochemical trends |
| 5 | appetite and nutrition strategy | Patient appetite |
| 5 | familiarity with product | Clinician preference |
| 3 | Goal to avoid hypoglycemia at home (starting low on scale) | Risk of hypoglycemia |
| 3 | cost of NPH vs vetsulin | Cost and owner finances |
| 4 | Appetite | Patient appetite |
| 4 | primary DVM comfort with various insulins | Primary care veterinarian preferences |
| 5 | I often choose what the primary veterinarian has available for dogs as they will be doing the follow up | Primary care veterinarian preferences |
| 5 | low cost generic | Cost and owner finances |
| 5 | Whether there's a strong concern for hypoglycemia with bolus insulins (e.g. a patient who is a picky/not reliable eater) | Patient appetite |
| 3 | Highest BG | In-hospital patient biochemical trends |
| 5 | Availability (of insulin) | Insulin characteristics |
| 3 | Comorbidities present | Patient factors |
| 5 | Owner schedule needs | Miscellaneous |
| 5 | how does on first 12 hours of intermediate insulin | In-hospital patient biochemical trends |
| 2 | Cost of insulin | Cost and owner finances |
| 4 | Patient eating/not having massive losses from vomiting/diarrhea prior to starting long/intermediate acting insulin | Patient appetite |
| 3 | Bglu at time of administration, rechecked off of the insulin CRI | In-hospital patient biochemical trends |
| 5 | pen formulation | Insulin characteristics |
| 5 | CGM use at discharge vs. not | Diabetic monitoring strategy |
| 5 | Patient behavior and tolerance for injections | Patient factors |

Note: Comments were taken directly from participant responses and no editing was performed.

**Supplementary material.** Free-text comments in response to the question, “Please share any additional comments below”; (n=10).

| **Comment** | **Code or Theme** |
| --- | --- |
| Our hospital generally uses an intermittent SQ DKA protocol, so CRI totals I do not usually use at all when deciding rather, response to intermittent injections. This protocol is often used a. because it seems to be non-inferior and b. because it is easier for nursing staff. | In-hospital management of DKA |
| Would love to learn more about how to use the CRI/total daily dose received to determine initial dose! (If that is relevant) | Desire for more clear guidelines regarding insulin dosing |
| I transition to a standard dose of intermediate or long acting insulin but supplement with regular insulin if glucose goes above 350mg/dl. The need for supplemental regular insulin guides the subsequent days insulin dose. I am considering transitioning DKA dogs to once daily insulin degludec but cost and availability of the biosimilar has been an issue. | Transition to an intermediate or long-acting insulin |
| You used two small patients for the example. What about a 45kg Rottweiler? Some additional clarity might be apparent about starting doses in large patients. One of the selections was not "label recommendations". ProZinc and Vetsulin have starting doses on the label. Everything else is off label. | Feedback on the survey itself |
| As a specialist I probably would have chosen Degludec in the first case if we had been given more owner factors such as how many times they would have preferred. I generally do not use intermediate acting insulins anymore. I also regret not ranking type of monitoring being used post-hospitalization as critically important for my insulin choice--- If I have a libre I am more bold, If I am doing fructosamine alone in a fractious cat I am preferring safety- ie lowest risk of hypoglycemia (Toujeo if not Senvelgo) | Transition to an intermediate or long-acting insulin |
| I typically begin long acting insulin in combination with CRI therapy. Additionally, I usually keep DKA patients in hospital on long acting insulin for at least 24 hours before discharge to attempt to titrate an appropriate dose. | Transition to an intermediate or long-acting insulin |
| I also include patients eating habits within hospital and if it may differ at home, type of food, amount eating, etc. if not eating “great” but enough- then i may start a little lower. | Transition to an intermediate or long-acting insulin |
| I typically add dextrose to IV fluids when patients are on insulin CRIs to target BGs in the 150-250 mg/dl range. I didn't see dextrose supplementation noted, assuming this was not given, and I typically factor need for dextrose while on the CRI in to my post-CRI dosing plan. I'm also curious about the feline patient's CRI rate that was decreased as the patient because quite hyperglycemic. | In-hospital management of DKA |
| I've always found & observed the transition from insulin CRI to intermittent injections to be awkward & with little hard/scientific guidance as to what TO DO & what NOT TO DO. | Desire for more clear guidelines regarding insulin dosing |
| Tried to go back to dog case to correct insulin dosage as 0.25-0.3 u/kg or to calculate total dose but could not go back to correct the entry | Feedback on the survey itself |

Note: Comments were taken directly from participant responses and no editing was performed.
