# Supplementary material for: Factors influencing insulin prescribing practices among small animal specialists
Source: Front Vet Sci. 2026 May 21;13:1792480. doi: 10.3389/fvets.2026.1792480 (PMC13233279; doi:10.3389/fvets.2026.1792480)
Supplement: Supplementary file 3 [file Table_3.docx]

​​​​**Case 1:** Nala

Nala, a 14-year-old female spayed Poodle Mix, was presented to a tertiary referral hospital for suspected diabetic ketoacidosis (DKA).

**Weight:** 4.65 kg
**Vitals on presentation:** T 100.6F, P 100, R 24
**Physical examination abnormalities:** 5-7% dehydrated, pale pink mucous membranes, moderate cranial abdominal pain

Initial blood work revealed a blood glucose level of 588 mg/dL (normal = 75-126 mg/dl), blood ketone levels of 4.8 mmol/L (normal = <3.8 mmol/L), blood pH of 7.29 (normal = 7.35-7.45), and blood bicarbonate levels of 15.4 mmol/L (normal = 20.7-29.2 mmol/L). Nala was diagnosed as a first-time diabetic in DKA based on the combination of the following findings: hyperglycemia, ketonemia, and a metabolic acidosis. A regular insulin CRI was initiated at 0.05 U/kg/hr.

Additional information regarding this patient's clinical picture can be found at the following link: [Nala](https://ncsu.qualtrics.com/CP/File.php?F=F_1Hr16kphQ0o0ugu)

Nala was hospitalized for ongoing management of DKA. Her regular insulin CRI was discontinued with an average blood glucose level of 145 mg/dL in the last 6 hours of administration.

In the last 24 hours of insulin administration prior to discontinuation, her average insulin CRI rate was 0.12 U/kg/hr. Her total daily insulin dose in the last 24 hours of administration was 2.9 U/kg, which equates to approximately 13.5 U total.

Nala – Additional Information

History:

Nala, a 14-year-old female spayed Poodle Mix, was presented to a tertiary referral hospital for evaluation of a 2-week history of hyporexia. Her past medical history includes myxomatous mitral valve disease (MMVD) Stage B1, tracheobronchial chondromalacia, and a chronic cough well-controlled on prednisone.

Current medications: Prednisone 2.5 mg PO q24h (~0.5 mg/kg) Diet: “Answers” raw diet; chicken and rice for the past 2 weeks Lifestyle: No travel history.

Full physical examination on presentation:

**Weight:** 4.65 kg

**Vitals:** T 100.6F, P 100 bpm, R 24 bpm

**General appearance:** QAR. 5-7% dehydrated (loss of skin elasticity, tacky mucous membranes).

**Ophthalmic:** Clear corneas with no ocular discharge. Lenticular opacity OU.

**Otic:** No aural debris or erythema AU.

**Nasal:** Mild nasal discharge of the right naris.

**Oral:** Severe dental disease. No ulceration, masses or foreign bodies.

**Cardiovascular:** Grade II-III/VI left apical systolic murmur. Normal heart rate and rhythm. Strong, synchronous pulses. CRT 1-2 seconds with pale pink mucous membranes.

**Respiratory:** Moderate referred upper airway noise during inhalation. No crackles or wheezes.

**Abdomen/gastrointestinal:** Moderate repeatable cranial abdominal pain on palpation. No palpable masses or fluid wave appreciated.

**Urogenital:** No vulvar discharge or mammary masses noted.

**Musculoskeletal:** Normal muscle mass. No pain on long bone palpation. No lameness. **Integumentary:** Mild urine scald in inguinal areas with solitary pustule in right inguinal region. Soft, freely moveable subcutaneous mass along sternum on midline.

**Lymphatic:** No peripheral lymphadenopathy.

**Neurologic:** Normal mentation. No ataxia. No palpebral reflex and menace response intact OU.

**Rectal:** Not examined.

Initial diagnostics on presentation:

1. Point-of-care blood work: BG 588, PCV 25%, TS 8.7, Lac 1.6

1. Blood ketones: 4.8 mmol/L
2. Venous blood gas: pH 7.29, pCO2 32, pO2 53, Na 136, K 4.0, Cl 103, iCa 1.33, Glu

394, Lac 1.8, TCO2 16.4, BE -10.2, HCO3 15.4, Hct 26

1. Complete blood count: WBC 18.59 (H), Hct 21.2 (L), MCV 74.0, MCHC 31.4, Abs

retic 44000, Plt 503 (H), Segs 15.430 (H), Bands 0.558, Lymph 1.115, Mono 0.558

1. Chemistry panel: Glu 152 (H), BUN 71 (H), Creat 1.7 (H), P 9.0 (H), Ca 8.8 (L), Mg 2.3,

TP 5.4, Alb 3.4, Glob 2.0, Chol 311, Tbili <0.2, ALP 242 (H), ALT 50, AST 55 (H), GGT

11 (H), CK 261 (H), Na 143 (L), K 4.4, Cl 109, Bicarb 14 (L), AG 24.7, Amy 1215 (H),

Lip 1077 (H), CRP 71.4 (H)

1. Urinalysis: pH 6.0, USG 1.020, 2+ protein, 4+ glucose, 2+ ketones, 2-5 coarse granular casts
2. Urine culture: no growth
3. Abdominal ultrasound conclusions:
   1. Cranial abdominal steatitis. Scant volume of cranial abdominal peritoneal fluid. Equivocal pancreatomegaly. This combination of findings is most consistent with pancreatitis and secondary regional peritonitis, as clinically suspected.
   2. Similar hyperechoic and nodular hepatopathy. This continues to be a nonspecific finding that may be secondary to metabolic disease (e.g. Cushing's or diabetes mellitus), regenerative change such as nodular hyperplasia, hepatitis (e.g. inflammatory or infectious), or infiltrative neoplasia such as lymphoma.
   3. Similar, diffusely mottled spleen. This is a nonspecific finding and may represent regenerative change such as lymphoid hyperplasia or extramedullary hematopoiesis. Infiltrative neoplasia such as lymphoma cannot be excluded.
   4. Similar left adrenal hyperechoic foci. Multifocal regions of fibrosis or fatty infiltration continued to be prioritized.
   5. Mild left renal pelvic dilation, otherwise similar bilateral chronic nonspecific nephropathy. The newly identified mild left renal pyelectasia is most consistent with current intravenous fluid therapy, however, progression of chronic renal disease or an acute on chronic nephropathy cannot be excluded.
   6. Similar, moderate volume of cholecystic debris.

Initial therapeutics given in hospital:

1. IV fluids: 0.45% NaCl 60 mL/kg/day
2. IV fluid additives: KCl 0.05 mEq/kg/hr, KPhos 0.05 mEq/kg/hr, Famotidine CRI 8 mg/kg/day
3. Enteral water: 7% replacement over 24 hours
4. Insulin CRI 0.05 U/kg/hr
5. GI support: Cerenia 1 mg/kg IV q24h, Ondansetron 0.5 mg/kg IV q8h, Sucralfate 0.5 g PO q6h
6. Anxiolysis & analgesia: Simbadol 0.015 mg/kg IV q8h, Gabapentin ~5 mg/kg PO q8h PRN, Trazodone ~5 mg/kg PO q8h PRN
7. Other: Dexamethasone SP 0.07 mg/kg IV q24h

*Graph 1: Patient’s regular insulin CRI rates and respective blood glucose and blood ketone levels in the final 24 hours of regular insulin CRI administration. Patient was initially started on a regular insulin CRI of 0.05 U/kg/hr at the start of hospitalization (not included on this graph).*
