# Supplementary material for: Factors influencing insulin prescribing practices among small animal specialists
Source: Front Vet Sci. 2026 May 21;13:1792480. doi: 10.3389/fvets.2026.1792480 (PMC13233279; doi:10.3389/fvets.2026.1792480)
Supplement: Supplementary file 4 [file Data_Sheet_1.pdf]

Consent Form

Title of Study: Insulin Prescribing Patterns of Veterinary Specialists

IRB Protocol: 27615

Principal Investigator(s): Katie McCool; kemccool@ncsu.edu

Funding Source: N/A

Collaborating Researchers: Adam Birkenheuer; ajbirken@ncsu.edu

---

You are being asked to complete a survey for research purposes. This survey is about insulin prescribing patterns for veterinary specialists. This survey will take roughly 10 minutes to complete.

You must be 18 years of age or older and live in the United States to participate in this study.

For this study, you will complete a survey related to your insulin prescribing patterns. Completing this survey is voluntary and you can stop at any time by closing your internet browser. If you decide to withdraw from this study after submitting the survey, you can do so by e-mailing Dr. Katie McCool kemccool@ncsu.edu.

We suggest that you take this survey using a private device, in a private location, using a web browser set to private/incognito mode. The data collected about you from this survey will be stored in accordance with NC State data protection standards.

You will not receive any payment for completing this survey.

If you have any questions about the survey, how it is implemented, or the research study, please contact the PI, Dr. Katie McCool at kemccool@ncsu.edu. Please reference study number 27615 when contacting anyone about this project.

If you have questions about your rights as a participant or are concerned with your treatment throughout the research process, please contact the NC State University IRB Director at IRB-Director@ncsu.edu, 919-515-8754, or fill out a confidential form online at https://research.ncsu.edu/administration/compliance/research-compliance/irb/irb-forms-and-templates/participant-concern-and-complaint-form/

If you consent to complete this survey, please select the "I consent" option below. If you do not consent to complete this survey, please select the "I do not consent" option below.

- ○ I consent
- ○ I do not consent

Introduction

The following case information was collected from a tertiary referral hospital. Because the current veterinary literature does not provide overt guidelines for transitioning

from a regular insulin CRI to intermediate or long-acting SQ insulin injections, the main objective of this survey is to gather clinicians' perspectives on this topic. There is no correct answer to each case example.

Thank you in advance for participating in our survey!

Case 1 Block

Case 1: Nala

Nala, a 14-year-old female spayed Poodle Mix, was presented to a tertiary referral hospital for suspected diabetic ketoacidosis (DKA).

Weight: 4.65 kg
Vitals on presentation: T 100.6F, P 100, R 24
Physical examination abnormalities: 5-7% dehydrated, pale pink mucous membranes, moderate cranial abdominal pain

Initial blood work revealed a blood glucose level of 588 mg/dL (normal = 75-126 mg/dl), blood ketone levels of 4.8 mmol/L (normal = <3.8 mmol/L), blood pH of 7.29 (normal

= 7.35-7.45), and blood bicarbonate levels of 15.4 mmol/L (normal = 20.7-29.2 mmol/L). Nala was diagnosed as a first-time diabetic in DKA based on the combination of the following findings: hyperglycemia, ketonemia, and a metabolic acidosis. A regular insulin CRI was initiated at 0.05 U/kg/hr.

Additional information regarding this patient's clinical picture can be found at the following link: Nala

Nala was hospitalized for ongoing management of DKA. Her regular insulin CRI was discontinued with an average blood glucose level of 145 mg/dL in the last 6 hours of administration.

In the last 24 hours of insulin administration prior to discontinuation, her average insulin CRI rate was 0.12 U/kg/hr. Her total daily insulin dose in the last 24 hours of administration was 2.9 U/kg, which equates to approximately 13.5 U total.

At this time, you choose to transition this patient from a regular insulin CRI to intermediate or long-acting SQ insulin injections. Which of the following options would you select for this patient?

- NPH (Humulin N, Novolin N)
- Lente (Vetsulin)
- PZI (ProZinc)
- Insulin glargine 100 U/mL (Lantus)
- Degludec (Tresiba)
- Insulin glargine 300 U/mL (Toujeo)
- Other:

At what frequency would you prescribe these injections?

- q8h
- q12h
- q24h

At what dose (in total units per injection) would you start initial SQ insulin therapy?

Case 2

Case 2: Bart

Bart, a 4-year-old male neutered Domestic Shorthair, was presented to a tertiary referral hospital for newly diagnosed diabetes mellitus, DKA and hyporexia.

Weight: 5.88 kg
Vitals on presentation: T 101.0F, P 190, R 30
Physical examination abnormalities: quiet to dull mentation, ~5% dehydrated

Initial blood work revealed a blood glucose level of 324 mg/dL (normal = 69-149 mg/dl), blood ketone levels of 4.4 mmol/L (normal = <2.55 mmol/L), blood pH of 7.09 (normal = 7.24-7.40), and blood bicarbonate levels of 11.2 mmol/L (normal = 15.6-26.5 mmol/L), confirming that Bart was in DKA. A regular insulin CRI was initiated at 0.05 U/kg/hr.

Additional information regarding Bart's case can be found at the following link: Bart

Bart was hospitalized for ongoing management of DKA. His regular insulin CRI was discontinued with an average blood glucose level of 280 mg/dL in the last 6 hours of administration.

In the last 24 hours of regular insulin administration prior to discontinuation, his average insulin CRI rate was 0.2 U/kg/hr. His total daily insulin dose in the last 24 hours of administration was 5 U/kg, which equates to approximately 29 U total.

At this time, you choose to transition this patient from a CRI of regular insulin to SQ injections of intermediate or long-acting insulin. Which of the following insulin options would you select for this patient?

- NPH (Humulin N, Novolin N)
- Lente (Vetsulin)
- PZI (ProZinc)
- Insulin glargine 100 U/mL (Lantus)

- Degludec (Tresiba)
- Insulin glargine 300 U/mL (Toujeo)
- Other:

At what frequency would you prescribe these injections?

- q8h
- q12h
- q24h

At what dose (in units per injection) would you start initial insulin therapy?

Reasoning for prescribing patterns

Given your previous selection of an initial insulin dosage and type for the two case examples, the following questions will ask about the reasoning and influences for those prescribing patterns.

Which factors influenced your decision in selecting an initial dose of intermediate or long-acting insulin? Please rate the importance of the following factors on a scale from "not at all important-1" to "very important-5".

	Not at all important	Slightly important	Moderately important	Important	Very important
	1	2	3	3	4	5
Personal standard dose for initiation of insulin therapy					
Literature-based standard dose for initiation of insulin therapy					
Insulin CRI data provided					
Total daily dose of insulin in last 24 hours of insulin CRI administration					
Body condition score of patient					
Comorbidities of patient					

	Not at all important	Slightly important	Moderately important	Important	Very important	
	1	2	3	3	4	5
Physical examination abnormalities of patient	○					☐
Blood work abnormalities of patient	○					☐
Patient's new diagnosis of diabetes mellitus	○					☐
Owner factors (e.g., ease of administration)	○					☐
Type of insulin selected	○					☐
Species	○					☐

Please rate the importance of any additional factors (not listed above), on a scale from "not at all important-1" to "very important-5":

	Not at all important 0	1	Slightly important 1	2	Moderately important 2	3	3	Important 4	4	Very important 5	5
Other (specify)	○										
Other (specify)	○										
Other (specify)	○										

If you rated "personal standard dose for initiation of insulin therapy" as important or very important, at what point in your training was this standard taught?

- ☐ During veterinary school as part of curriculum
- ☐ During veterinary school while in clinical rotations from personal experience
- ☐ During internship from personal experience
- ☐ During internship from a single mentor
- ☐ During internship from advice assimilated from multiple mentors
- ☐ During residency from personal experience
- ☐ During residency from a single mentor
- ☐ During residency from advice assimilated from multiple mentors
- ☐ Post-residency from personal experience
- ☐ Post-residency from a single mentor

- [ ] Post-residency from advice assimilated from multiple mentors
- [ ] Learned from literature
- [ ] N/A - this was not a factor in my insulin dosage determination
- [ ] _____ Other (specify)

If you rated "standard dose for initiation of insulin therapy" as important or very important, how consistently do you apply this standard in practice?

	0	10	20	30	40	50	60	70	80	90	100	
Percent of the time												

Demographic Questions

Of which college are you currently a diplomate?

- [ ] ACVIM-SAIM
- [ ] DACVECC
- [ ] _____ Other

How long have you been in practice as a veterinarian?

- 1-5 years
- 6-10 years
- 11-15 years
- 16-20 years
- 21-25 years
- 26-30 years
- 31-35 years
- 36-40 years
- 41-45 years
- >46 years

In which country do you currently practice?

- United States of America
- Canada
- Other:

In which hospital setting do you currently practice?

- Non-profit private practice
- For-profit private practice
- Academic institution

Other:

Please share any additional comments below.

Powered by Qualtrics
